# Higher serum lectin-like oxidized low-density lipoprotein receptor-1 in patients with stable coronary artery disease is associated with major adverse cardiovascular events: A multicentre pilot study

**DOI:** 10.11613/BM.2019.010705

**Published:** 2019-02-15

**Authors:** Zi-wen Zhao, Yi-wei Xu, Shu-mei Li, Jin-jian Guo, Tao Yi, Liang-long Chen

**Affiliations:** 1 Department of Cardiology, Union Hospital, Fujian Medical University, Fuzhou, P. R. China; 2 Fujian Institute of Coronary Artery Disease, Fuzhou, P. R. China; 3 Department of Cardiology, The 476 Clinical Department of Fuzhou General Hospital, Fuzhou, P. R. China; 4 Department of Cardiology, The Second People’s Hospital of Fujian Province, Fujian University of Traditional Chinese Medicine, Fuzhou, P. R. China

**Keywords:** coronary artery disease, sLOX-1, prognosis, major adverse cardiovascular events, biomarker

## Abstract

**Introduction:**

Lectin-like oxidized low-density lipoprotein receptor-1 (LOX-1) is involved in the pathophysiology of atherosclerosis and acute coronary syndromes (ACS). Circulating soluble LOX-1 (sLOX-1) has been linked to the risk of coronary artery disease (CAD). Our aim was to test if baseline serum sLOX-1 was associated with major adverse cardiovascular events (MACE) in patients with stable CAD.

**Materials and methods:**

This multicentre pilot study enrolled 833 stable CAD patients. All patients were followed for two years. Serum sLOX-1 concentrations were detected by enzyme-linked immunosorbent assay (ELISA). The association between sLOX-1 concentrations and MACE was assessed by logistic regression, Kaplan-Meier survival curves and Cox proportional hazards analyses. Logistic regression analysis was employed to assess the predictors of complex lesion.

**Results:**

Multivariate logistic regression analysis revealed that sLOX-1 concentration was an independent predictor of MACE (OR 2.07, 95%CI 1.52 - 2.82; P < 0.001). Kaplan-Meier cumulative survival curves showed that the incidence of MACE in patients with a high sLOX-1 concentration was significantly higher than in patients with an intermediate or low sLOX-1 concentration (P < 0.001). Soluble LOX-1 concentrations were independently correlated with coronary complex lesions (OR 2.32, 95%CI 1.81 - 2.97; P < 0.001).

**Conclusions:**

Baseline sLOX-1 concentrations were correlated with 2-year MACE in stable CAD patients. Furthermore, patients with high serum sLOX-1 concentrations had higher cumulative incidence of MACE compared to those with low serum sLOX-1 concentrations.

## Introduction

Coronary artery disease (CAD) remains the leading cause of mortality and disability worldwide (*1*). Stable CAD, defined as obstructive or non-obstructive CAD with stable symptoms, affects approximately 54 million patients worldwide and can vary substantially from a clinically stable condition to acute coronary syndrome (ACS) (*2*-*4*). Therefore, early identification of stable patients at high-risk is essential for proactive secondary prevention of cardiovascular (CV) events.

Symptomatic stability does not always mean coronary plaque stability in stable CAD patients. Vulnerable plaque rupture, causing intraluminal thrombus occluding the vessel lumen, accounts for the most devastating results of stable CAD: ACS and sudden death (*5*). Because stable CAD can take years to develop and has an unpredictable trajectory, dynamic risk assessment is a critical issue for the development of preventive strategies. However, monitoring stable CAD patients dynamically with computed tomography or invasively with coronary angiography (CAG) is expensive and sometimes has risk of harm (*6*). Besides, clinical risk factors and scores for predicting CV events often lack sufficient sensitivity and specificity (*7*). Therefore, reliable non-invasive blood biomarker might contribute to dynamic assessment of stable CAD in a cost-effective way.

Lectin-like oxidized low-density lipoprotein receptor-1 (LOX-1) is a class E scavenger receptor that primarily binds oxidized low-density lipoprotein (ox-LDL) (*8*). Evidence suggests that LOX-1 plays a crucial role in various steps of the atherosclerotic process including endothelial dysfunction; atherogenesis and plaque rupture (*9*). The pivotal role of LOX-1 in pathophysiology of atherosclerosis makes it a potential therapeutic target for CAD. The extracellular domain of LOX-1 might be cleaved proteolytically and released into the bloodstream as a soluble form, which is known as soluble LOX-1 (sLOX-1) (*10*). Like many membrane-bound proteins, circulating sLOX-1 concentrations could reflect the expression of LOX-1 (*11*). Circulating sLOX-1 has been used as a biomarker of ACS, diabetes mellitus (DM) and stroke (*12*-*16*). However, the relationship between sLOX-1 and long-term clinical outcome in stable CAD patients has not yet been fully investigated. Therefore, we conducted this study in a stable CAD cohort to investigate whether an association exists between baseline serum sLOX-1 concentration and major adverse cardiovascular events (MACE).

## Materials and methods

### Study population

From October 2012 to September 2015, 833 consecutive patients from the Cardiology Department of 3 superior hospitals in Fujian Province (Union Hospital Affiliated to Fujian Medical University, The Second People’s Hospital of Fujian Province and The 476 Clinical Department of Fuzhou General Hospital) were enrolled. These patients did not participate in any other studies.

### Study protocol

**Figure 1:**
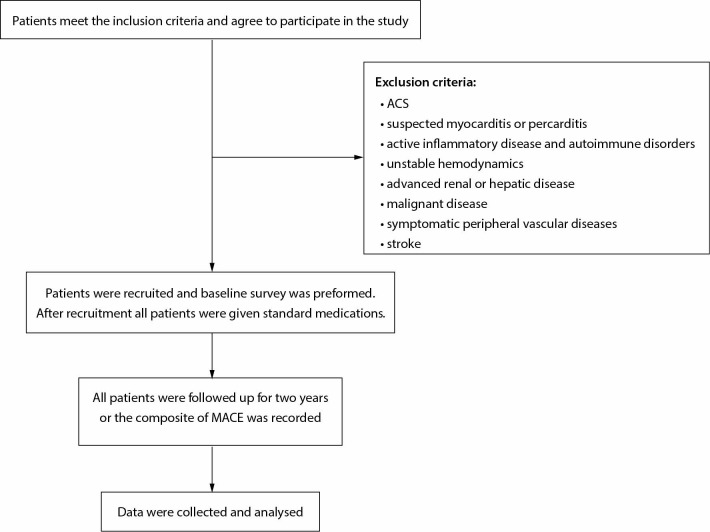
The flowchart of study protocol. ACS - acute coronary syndrome. MACE - major adverse cardiovascular events.

The flowchart of study protocol is shown in Figure 1. We have chosen a multicentre observational study design and recruited patients with no rest chest pain or recent deterioration for at least three months before study entry, but with angiographically documented primary coronary artery stenosis or stenosis of its major branches greater than 50%. Participants were excluded from the present study if they had ACS, suspected myocarditis or pericarditis, active inflammatory disease and autoimmune disorders, unstable haemodynamics, advanced renal or hepatic disease, malignant disease, symptomatic peripheral vascular diseases or stroke. Acute coronary syndrome included acute myocardial infarction (AMI) and unstable angina (UA). Acute myocardial infarction was defined as a distinct clinical event, with either: (*1*) increase in troponin I (Troponin I was measured upon admission and repeated two or more times over the next 6 to 24 hours) ≥ 2 times the upper limit of normal range; (*2*) development of new Q waves or significant ST-segment elevation or depression in ≥ 2 contiguous electrocardiogram leads; (*3*) new left branch bundle block pattern (*17*). Unstable angina was diagnosed according to Braunwald (*18*).

Traditional cardiovascular risk factors including hypertension, DM and smoking status were included in the statistical analyses to appreciate sLOX-1 uniqueness. Hypertension was defined as systolic blood pressure (SBP) ≥ 140 mmHg or diastolic blood pressure (DBP) ≥ 90 mmHg, or recently taking medication for hypertension. Diabetes mellitus was defined as fasting blood glucose (FBG) value ≥ 7.0 mmol/L or non-fasting blood glucose ≥ 11.1 mmol/L and/or any hypoglycaemic medication taken at admission. Smoking status was defined as more than one cigarette a day and lasting at least half a year.

At study entry, a baseline survey including age, gender, body mass index (BMI), blood pressure, cardiovascular risk factors and cardiovascular medication was performed. Cardiac functional status was classified according to the New York Heart Association (NYHA) functional classification.

After recruitment, all patients were followed up for two years. During the 2-year follow-up period, patients were given standard medications including anti-platelet agents, statins, angiotensinogen converting enzyme inhibitors (ACEI)/angiotensin receptor blockers (ARB) and beta blocker according to up-to-date guidelines. The primary end-point of this study was the composite of MACE, which were identified as all-cause death, nonfatal AMI and readmission for Braunwald’s class IIIb UA requiring treatment. Hospital readmissions and deaths were identified by electronic patient records and through direct contact with the patients or relatives.

All patients have provided written informed consent. The study protocol was approved by the institutional ethics committee.

### Methods

Dimensional echocardiogram of the patients was measured using GE Vivid 3 cardiovascular ultrasound equipment (General Electrics, Milwaukee, USA).

Venous blood samples were drawn in a fasting state under standardized conditions (blood samples were collected from basilic vein into plain biochemistry tubes). After clotting, blood samples were centrifuged at 3000 rounds *per* minute (rpm) for 15 minutes. The serum samples were stored at - 80 °C until analysis. Routine laboratory parameters including FBG, triglycerides (TG), total cholesterol (CHOL), low density lipoprotein cholesterol (LDL), high density lipoprotein cholesterol (HDL), creatinine (CREA), urea and uric acid (UA) were measured using standard laboratory techniques by automatic biochemical analyser (LX-20; Beckman Coulter, Brea, USA). N-terminal pro-brain natriuretic peptide (NT-proBNP) concentrations were determined on Cobas 6000 analyser series (F. Hoffmann-La Roche Ltd; Basel, Switzerland). High-sensitivity C-reactive protein (hs-CRP) concentrations were determined on Immage 800 Immunochemistry System (Beckman Coulter, Brea, USA). Homocysteine (Hcys) concentrations were determined on Abbott i2000SR analyser (Abbott, Abbott Park, USA). Circulating sLOX-1 concentrations was measured using an enzyme-linked immunosorbent assay (ELISA) kit with an intra-assay and inter-assay CV < 5% according to a published experiment (USCN, Wuhan, China) (*19*). All ELISA determinations were routinely performed in duplicate. The results were averaged to minimize measurement errors.

All patients underwent CAG, which was performed in the catheterization room according to standard protocols. Angiograms were analysed by two experienced interventional cardiologists blinded to the study protocol. Coronary lesion morphology was grouped into simple or complex lesion according to the Ambrose classification (*20*, *21*).

### Statistical analyses

The study sample size was calculated using power analysis with the following assumptions: an expected MACE rate of 10% in stable CAD patients, a 0.5 ng/mL difference in mean sLOX-1 concentration between outcome groups, using an alpha error of 0.05 and power of 0.9. Therefore, at least 47 outcome events were needed (*22*). Data distribution patterns were tested by the Kolmogorov-Smirnov test. Continuous variables were presented as mean ± standard deviation (SD) or median (interquartile range). Categorical and ordinal variables were presented as numbers and percentages. Differences between the two groups were analysed with the unpaired t-test, Mann-Whitney U test, Chi-square or Fisher’s exact text as indicated. Correlations between variables and sLOX-1 concentrations were analysed by Spearman correlation analysis. Univariate and multivariate logistic regression was employed to assess the predictors of MACE. Kaplan-Meier analysis with log-rank test was performed to compare the survival curves for sLOX-1 concentrations in different tertiles. Cox proportional hazard analyses were used to evaluate the association between sLOX-1 concentrations in different tertiles and MACE after adjustment for potential confounding factors. Logistic regression was performed to assess the predictors of complex lesion. For all regression analyses, univariate factors with P values < 0.05 entered the stepwise backward multivariate regression analysis. All analyses were performed by using SPSS 22.0 software (SPSS Inc., Chicago, USA). For all analyses, P values less than 0.05 (two-tailed) were required to reject the null hypothesis.

## Results

### Baseline characteristics and sLOX-1 concentrations

As shown in Table 1, 75 patients (9%) suffered the combined end-points, whereas 758 patients had no events. Baseline characteristics and sLOX-1 concentrations of patients with and without MACE are shown in Table 2. The relationship between sLOX-1 concentrations and various sociodemographic and cardiovascular risk factors is presented in Table 3 and Table 4.

**Table 1:** Major adverse cardiovascular events in the follow-up period

**Event**	**N (%)**
All-cause death	6 (0.72)
Nonfatal AMI	17 (2.04)
Class IIIb unstable angina	52 (6.24)
Total	75 (9.00)
The study group consisted of 833 patients. AMI - acute myocardial infarction.	

**Table 2:** Baseline characteristics of patients

	**No MACE** **(N = 758)**	**MACE** **(N = 75)**	**P**
Age (years)	64 (32 - 87)	68 (39 - 84)	0.009
Male, N (%)	573 (76)	54 (72)	0.491
BMI (kg/m^2^)	24 (24 - 26)	24 (23 - 26)	0.135
SBP (mmHg)	130 (120 - 145)	130 (120 - 148)	0.801
DBP (mmHg)	80 (70 - 85)	80 (70- 90)	0.893
FBG (mmol/L)	5.4 (5.0 - 6.2)	5.9 (5.1 - 7.5)	0.010
TG (mmol/L)	1.4 (1.0- 2.0)	1.3 (1.0 - 1.9)	0.723
CHOL (mmol/L)	4.1 (3.4 - 5.1)	4.3 (3.7 - 5.3)	0.040
LDL (mmol/L)	2.5 (1.9 - 3.4)	2.9 (2.0 - 3.8)	0.052
HDL (mmol/L)	1.1 (0.9 - 1.3)	1.1 (0.9 - 1.3)	0.986
urea (mmol/L)	4.9 (4.1 - 5.9)	6.0 (4.5 - 7.1)	< 0.001
CREA (μmol/L)	77 (67 - 89)	80 (69 - 95)	0.101
UA (μmol/L)	366 (310 - 431)	365 (316 - 424)	0.793
NT-proBNP (ng/L)	75 (36 - 191)	203 (71 - 943)	< 0.001
hs-CRP (mg/L)	1.1 (0.5 - 2.8)	1.7 (0.7 -4.3)	0.038
Hcys (μmol/L)	9.2 (7.6 -11.2)	9.4 (8.4 - 11.4)	0.290
sLOX-1 (ng/mL)	0.58 (0.39 - 1.01)	1.04 (0.69 - 1.80)	< 0.001
**Cardiovascular risk factors**			
Smoking, N (%)	385 (51)	34 (45)	0.522
DM, N (%)	228 (30)	34 (45)	0.007
Hypertension, N (%)	489 (65)	54 (72)	0.194
Previous AMI, N (%)	145 (19)	18 (24)	0.310
LVEF (%)	65.8 (60.3 - 70.3)	62.0 (48.2 - 67.0)	< 0.001
NYHA (III or IV), N (%)	48 (6)	25 (33)	< 0.001
**Cardiovascular medication**			
Statins, N (%)	739 (98)	73 (97)	0.933
ACEI/ARB, N (%)	533 (70)	50 (67)	0.511
Beta-blocker, N (%)	551 (73)	54 (72)	0.898
**Coronary angiography**			
Number of diseased vessels > 50%, N (%)	2 (1 - 2)	2 (1 - 3)	< 0.001
Complex lesion, N (%)	173 (23)	41 (55)	< 0.001
PCI, N (%)	574 (76)	52 (69)	0.222
CABG, N (%)	3 (0.39)	2 (2.67)	0.067
Age is presented as median (range), other values are presented as median value (interquartile range) or N (proportion). MACE - major adverse cardiovascular events. BMI - body mass index. SBP - systolic blood pressure. DBP - diastolic blood pressure. FBG - fasting glucose. TG – triglycerides. CHOL - total cholesterol. LDL - low density lipoprotein cholesterol. HDL - high density lipoprotein cholesterol. CREA – creatinine. UA - uric acid. NT-proBNP - N-terminal pro-brain natriuretic peptide. hs-CRP - high sensitive C reactive protein. Hcys – homocysteine. DM - diabetes mellitus. AMI - acute myocardial infarction. LVEF - left ventricular ejection fractions. NYHA - New York Heart Association functional class. ACEI - angiotensin converting enzyme inhibitor. ARB - angiotensin receptor blocker. PCI - percutaneous coronary intervention. CABG - coronary artery bypass grafting. sLOX-1 - soluble lectin-like oxidized low-density lipoprotein receptor-1. P < 0.05 was considered statistically significant.			

**Table 3:** Distribution of sLOX-1 according to various variables

**Variable**	**N**	**Serum sLOX-1 (ng/mL)**	**P**
**Sex**			
female	206	0.62 (0.41 - 1.05)	0.849
male	627	0.63 (0.43 - 1.01)	
**Smoking**			
Yes	407	0.58 (0.39 - 1.04)	0.338
No	426	0.63 (0.43 - 1.03)	
**DM**			
Yes	262	0.60 (0.39 - 1.13)	0.920
No	571	0.62 (0.42 - 1.02)	
**Hypertension**			
Yes	543	0.62 (0.42 - 1.03)	0.894
No	290	0.62 (0.40 - 1.03)	
**Previous AMI**			
Yes	163	0.61 (0.41 - 1.02)	0.085
No	670	0.70 (0.42 - 1.13)	
All values are presented as median value (interquartile range). sLOX-1 - soluble lectin-like oxidized low-density lipoprotein receptor-1. DM - diabetes mellitus. AMI - acute myocardial infarction. P < 0.05 was considered statistically significant.			

**Table 4:** Correlation between various variables and sLOX-1

**Variables**	**Correlation coefficient**	**P**
Age	0.03	0.353
BMI	- 0.04	0.305
SBP	- 0.01	0.734
DBP	- 0.02	0.606
FBG	0.02	0.669
TG	0.01	0.683
CHOL	- 0.05	0.196
LDL	- 0.04	0.259
HDL	0.01	0.747
urea	0.08	0.027
CREA	0.00	0.988
UA	0.01	0.884
NT-proBNP	0.01	0.783
hs-CRP	0.06	0.094
Hcys	0.02	0.626
sLOX-1 - soluble lectin-like oxidized low-density lipoprotein receptor-1. BMI - body mass index. SBP - systolic blood pressure. DBP - diastolic blood pressure. FBG - fasting glucose. TG – triglycerides. CHOL - total cholesterol. LDL - low density lipoprotein cholesterol. HDL - high density lipoprotein cholesterol. CREA – creatinine. UA - uric acid. NT-proBNP - N-terminal pro-brain natriuretic peptide. hs-CRP - high sensitive C reactive protein. Hcys - homocysteine. P < 0.05 was considered statistically significant.		

### Predictors of MACE in logistic regression

Predictors of MACE in univariate and multivariate logistic regression were shown in Table 5.

**Table 5:** Predictors of MACE

	**Univariate logistic regression**		**Multivariate logistic regression**	
**Variables**	**OR (95% CI)**	**P**	**OR (95% CI)**	**P**
Age (year)	1.03 (1.00 - 1.06)	0.010	1.01 (0.98 - 1.04)	0.622
FBG (mmol/L)	1.20 (1.08 - 1.32)	0.001	1.15 (1.00 - 1.34)	0.058
CHOL (mmol/L)	1.21 (1.01 - 1.44)	0.035	0.99 (0.54 - 1.82)	0.972
LDL (mmol/L)	1.22 (1.02 - 1.47)	0.034	1.24 (0.65 - 2.38)	0.515
urea (mmol/L)	1.24 (1.10 - 1.40)	< 0.001	1.11 (0.95 - 1.29)	0.201
NT-proBNP (ng/L)	1.00 (1.00 - 1.00)	0.039	1.00 (1.00 - 1.00)	0.533
DM (yes)	1.93 (1.19 - 3.12)	0.007	0.93 (0.48 - 1.80)	0.819
LVEF (1%)	0.94 (0.92 - 0.96)	< 0.001	0.98 (0.95 - 1.01)	0.139
NYHA (III or IV)	3.08 (2.23 - 4.26)	< 0.001	2.17 (1.40 - 3.36)	0.001
Number of diseased vessels >50% (yes)	1.72 (1.29 - 2.30)	< 0.001	1.34 (0.95 - 1.88)	0.093
Complex lesion (yes)	4.08 (2.51 - 6.62)	< 0.001	2.78 (1.60 - 4.83)	< 0.001
CABG (yes)	6.90 (1.13 - 41.93)	0.036	3.61 (0.29 - 44.29)	0.316
sLOX-1 (ng/mL)	2.51 (1.91 - 3.31)	< 0.001	2.07 (1.52 - 2.82)	< 0.001
MACE - major adverse cardiovascular events. OR - odds ratio. CI - confidence interval. FBG - fasting glucose. CHOL - total cholesterol. LDL - low density lipoprotein cholesterol. NT-proBNP - N-terminal pro-brain natriuretic peptide. DM - diabetes mellitus. LVEF - left ventricular ejection fractions. NYHA - New York Heart Association functional class. CABG - coronary artery bypass grafting. sLOX-1 - soluble lectin-like oxidized low-density lipoprotein receptor-1. P < 0.05 was considered statistically significant.				

### Survival analyses

**Figure 2:**
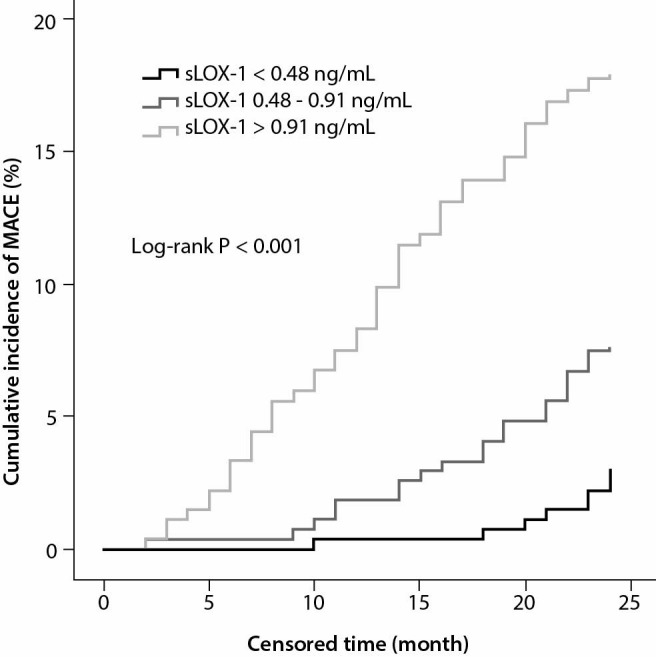
The cumulative incidence of MACE is depicted according to sLOX-1 distribution in tertiles. MACE - major adverse cardiovascular events. sLOX-1 - soluble lectin-like oxidized low-density lipoprotein receptor-1.

Cumulative incidence of MACE categorized by sLOX-1 distribution in tertiles is shown in Figure 2. The cumulative incidence of MACE was 16.61% in the high tertile of the sLOX-1 distribution (> 0.91 ng/mL) compared to 2.88% and 7.55% in the low (< 0.48 ng/mL) and intermediate tertile (0.48 - 0.91 ng/mL), respectively. The hazard ratios (HRs) and 95% confidence interval (CI) for development of MACE according to sLOX-1 concentrations as tertiles in different Cox proportional hazards models are presented in Table 6.

**Table 6:** Hazard ratios for MACE according to sLOX-1 concentrations in Cox proportional hazards models

**Variables**	**HR (95% CI)**			**P**
	**Tertile 1 (sLOX < 0.48 ng/mL)**	**Tertile 2 (sLOX 0.48 - 0.91 ng/mL)**	**Tertile 3 (sLOX > 0.91 ng/mL)**	
Model 1	1.0 (reference)	2.68 (1.19 - 6.06)	6.35 (3.00 - 13.45)	< 0 .001
Model 2	1.0 (reference)	2.59 (1.15 - 5.85)	6.20 (2.93 - 13.15)	< 0.001
Model 3	1.0 (reference)	2.54 (1.12 - 5.75)	6.20 (2.92 - 13.18)	< 0.001
Model 4	1.0 (reference)	2.75 (1.21 - 6.24)	4.73 (2.17 - 10.30)	< 0.001
Model 1: Non-adjusted model. Model 2: adjusted for age and sex. Model 3: adjusted for age, sex and urea. Model 4: adjusted for age, sex, urea and NYHA functional class (III or IV) and complex lesion. HR - hazard ratio. CI - confidence interval. MACE - major adverse cardiovascular events. sLOX-1 - soluble lectin-like oxidized low-density lipoprotein receptor-1. P < 0.05 was considered statistically significant.				

### Predictors of the complex lesion in logistic regression

Predictors of the complex lesion in univariate and multivariate logistic regression are shown in Table 7.

**Table 7:** Predictors of complex lesion in stable CAD patients by logistic regression

	**Univariate logistic regression**		**Multivariate logistic regression**	
**Variables**	**OR (95% CI)**	**P**	**OR (95% CI)**	**P**
Previous AMI (yes)	1.70 (1.17 - 2.45)	0.005	1.16 (0.75 - 1.79)	0.499
LVEF (1%)	0.98 (0.97 – 1.00)	0.010	0.99 (0.97 - 1.01)	0.362
NYHA (III or IV)	2.64 (1.62 - 4.31)	< 0.001	1.22 (0.94 - 1.57)	0.131
Number of diseased vessels >50% (yes)	1.24 (1.02 - 1.50)	0.029	0.93 (0.75 - 1.15)	0.490
PCI (yes)	3.51 (2.21 - 5.60)	< 0.001	3.57 (2.16 - 5.89)	< 0.001
sLOX-1 (ng/mL)	2.41 (1.89 - 3.07)	< 0.001	2.32 (1.81 - 2.97)	< 0.001
AMI - acute myocardial infarction. LVEF - left ventricular ejection fractions. NYHA - New York Heart Association functional class. PCI - percutaneous coronary intervention. sLOX-1 - soluble lectin-like oxidized low-density lipoprotein receptor-1. OR - odds ratio. CI - confidence interval. P < 0.05 was considered statistically significant.				

## Discussion

The main findings of the present study are that in patients with stable CAD baseline serum sLOX-1 concentrations were independently correlated with 2-year MACE and patients with high serum sLOX-1 concentrations had significantly higher cumulative incidence of MACE compared to those with low serum sLOX-1 concentrations.

In our study, the overall incidence rate of MACE in stable CAD patients was in concordance with that reported in previous study involving Chinese populations (*23*). We demonstrated that patients who suffered from MACE had higher sLOX-1 concentrations compared to event-free patients. Lectin-like oxidized low-density lipoprotein receptor-1 is abundantly expressed in atherosclerotic lesions and rapidly induced by various pro-oxidative and pro-inflammatory stimuli (*9*). High concentrations of circulating sLOX-1 might reflect the increased expression of membrane-bound LOX-1 in patients suffering from MACE. This result is in accordance with previous studies suggesting that sLOX-1 concentrations were significantly higher in ACS patients and correlated with adverse clinical outcomes (*24*, *25*). It should be stated that no correlation was found between sLOX-1 and sociodemographic as well as cardiovascular risk factors. These results indicate that these potential confounding factors had no effect on the association between sLOX-1 and MACE.

In univariate logistic regression analysis, several factors were associated with MACE. Multivariate logistic regression revealed that sLOX-1 concentration was an independent predictor of MACE. As Class IIIb unstable angina and nonfatal AMI makes up most of the MACE, the association between sLOX-1 and future MACE is independent of traditional CV risk factors and indicates the possibility that sLOX-1 may represent a composite biomarker that reflects the complex process of CAD acute progression. Kaplan-Meier survival curves, based on the distribution of sLOX-1 concentrations, showed early and persistent separation during 2-year follow-up. Cox proportional hazards analysis confirmed the results observed in Kaplan-Meier survival curves. After adjustment for potential confounding factors, patients with high serum sLOX-1 concentrations still had higher risk of suffering MACE. These findings strengthen the potential role of sLOX-1 for identifying those at high-risk of future CV events in stable CAD patients.

Various studies have shown that angiographically complex lesion could provide prognostic information in CAD patients (*20*, *26*-*28*). We found that complex lesion was predictive of MACE in multivariate logistic regression analysis. We also showed that sLOX-1 concentration was independently associated with the presence of complex lesion in stable CAD patients. This result is in accordance with our previous study (*16*). These findings indicate that the correlation between increased risk of future MACE observed in patients with high sLOX-1 concentrations might be related to the increased prevalence of complex lesions.

Although this analysis was conducted in a large, well-characterized prospective patient cohort, the potential limitations of our study merit careful consideration. First, as with all non-randomized, observational studies, we cannot rule out that there was residual confounding present. Second, CAG provides insufficient information regarding true coronary lesion vulnerability. Updated vascular imaging modalities like optical coherence tomography (OCT) or intravascular ultrasound (IVUS) are needed to further evaluate the relationship between sLOX-1 and vulnerable plaques.

In conclusion, baseline sLOX-1 concentrations were correlated with 2-year MACE in stable CAD patients. If future studies prove the causality of the observation, sLOX-1 might emerge as a promising biomarker for risk stratification and a target for secondary prevention in these patients.
